# Analyzes of the ICF Domain of Activity After a Neurological Early Mobility Protocol in a Public Hospital in Brazil

**DOI:** 10.3389/fresc.2022.864907

**Published:** 2022-08-15

**Authors:** Fernanda dos Santos Lima, Vinícius da Silva Carvalho, Inaiacy Souto Bittencourt, Ana Paula Fontana

**Affiliations:** Functional Recovery Laboratory in Stroke, Department of Physiotherapy, Federal University of Rio de Janeiro, Rio de Janeiro, Brazil

**Keywords:** physical therapy, early mobility, wards, ICF, rehabilitation

## Abstract

**Background:**

Early Mobility (EM) has been recognized as a feasible and safe intervention that improves functional outcomes in hospitalized patients. The International Classification of Functioning, Disability and Health (ICF) supports understanding of functioning and disability in multidimensional concepts and efforts have been taken to apply ICF in a hospital environment. EM protocols might be linked with the ICF component of activity and participation. The correlations between ICF, EM, and functional scales might help the multidisciplinary team to conduct the best rehabilitation program, according to patients' functional demands.

**Objectives:**

The primary outcome is to analyze the activity level of neurological inpatients on admission and delivery after a Neurological Early Mobility Protocol (NEMP) at intermediate care settings in a public hospital in Brazil using Activity Level categories, HPMQ, and MBI scores. The secondary outcome is to analyze the ICF performance qualifier, specifically in the activity domain, transposing HPMQ and MBI scores to the corresponding ICF performance qualifiers.

**Design:**

An international prospective study.

**Methods:**

NEMP was used to promote patients' mobility during a hospital stay in neurological ward settings. First, patients were categorized according to their Activity Levels (ALs) to determine the NEMP phase to initiate the EM protocol. ALs also were evaluated in the first and last sessions of NEMP. Thereafter, the Hospitalized Patient Mobility Questionnaire (HPMQ) was applied to identify whether patients needed assistance during the performance of hospital activities as well as the Modified Barthel Index (MBI). Both measures were applied in NEMP admission and discharge, and the Wilcoxon Signed Rank Test was used to compare data in these two time points. HPMQ and MBI scores were re-coded in the correspondent ICF performance qualifier.

**Results:**

Fifty-two patients were included with age of 55 ± 20 (mean ± SD) years and a length of hospital stay of 33 ± 21 days. Patients were classified along ALs categories at the admission/discharge as follows: AL 0 *n* = 6 (12%)/*n* = 5 (9%); AL 1 *n* = 12 (23%)/*n* = 6 (12%); AL 2 *n* = 13 (25%)/*n* = 8 (15%); AL 3 *n* = 10 (19%)/*n* = 13 (25%); AL 4 *n* = 11 (21%)/*n* = 20 (39%). HPMQ data revealed progressions for the activities of bathing (*p* < 0.001), feeding (*p* < 0.001), sitting at the edge of the bed (*p* < 0.001), sit to stand transition (p < 0.001), orthostatism (*p* < 0.001) and walking (*p* < 0.001). Transposing HPMQ activities into ICF performance qualifiers, improvements were shown in bathing (d510.3 to d510.1—severe problem to mild problem) and sitting at the edge of the bed (d4153.2 to d4153.1—moderate problem to mild problem). At MBI score were observed an average of 36 [IQR−35. (95% CI 31.5; 41.1)] on NEMP admission to 52 at discharge [IQR−50 (95% CI 43.2; 60.3)] (*p* < 0.001). Recoding MBI scores into ICF there were improvements from severe problem (3) to moderate problem (2).

**Limitations:**

The delay in initiating NEMP compared to the period observed in the literature (24–72 h). The study was carried out at only one center.

**Conclusions:**

This study suggests that neurological inpatients, in a public hospital in Brazil had low activity levels as could be seen by MBI and HPMQ scores and in the ICF performance qualifier. However, improvements in the evaluated measures and ICF activity domain were found after NEMP. The NEMP protocol has been initiated much longer than 72 h from hospital admission, a distinct window than seen in the literature. This enlargement period could be a new perspective for hospitals that are not able to apply mobility in the earliest 24–72 h.

## Introduction

Patients with neurological diseases can also have disabilities that result in transient or permanent loss of functioning. Motor and sensory impairments, cognition decline, as well as differences in language or perception, mean many patients require assistance with the activities of daily living (1). In a hospital environment, this population are prone to bed rest and generally have limited mobility levels (2). For example, Bernhardt et al. (3) outline that patients rest in bed more than 50% of the time during a hospital stay. Early Mobility (EM) has been suggested as a powerful intervention that promotes a higher level of mobility, reduces the length of hospital stay, and enables them to achieve better functioning after discharge, as it allows independence during hospitalization (4, 5). Nurses and physiotherapists are the main professionals in the rehabilitation team who assess patients' motor activities and encourage mobility during a hospital stay (6).

Perme and Chandrashekar (7) defined EM in intensive care units as a mobility program that includes educational efforts, positioning care, mobility training in bed, and walking activities. They initiate EM when a patient has minimal ability to engage in therapy and a stable clinical status. In the same way, Drolet et al. (8) have constructed an EM protocol that progresses through the patient's mobility level, increasing the percentage of patients ambulating during the first 72 h of hospital stay.

Considering a stroke population, the timing for initiating EM is crucial for the effectiveness of this intervention (4). Bernhardt et al. (9) outline the timing of important biological processes in the cerebral tissue after stroke with implications for rehabilitation time. They propose a timeline of biological events and recovery potential since the hyperacute phase (0–24 h after stroke), acute (24 h−7 days), early subacute (7 days−3 months), late subacute (3–6 months) until the chronic phase after stroke (>6 months). EM trials usually investigate the repercussion of this approach in the first week after stroke, contemplating the recovery potential of brain plasticity.

EM in stroke units usually includes out-of-bed activities that can be initiated between 24 and 72 h. In this context, EM is related to the reduction of length of hospital stay, and independence in walking activity, with no occurrence of adverse events (10, 11). Otherwise, EM can also be initiated after the first 24 h of stoke (known as Very Early Mobilization–VEM) depending on the duration, frequency, and intensity of exercises, VEM can reduce the odds of favorable outcomes 90 days post stroke, as seen in AVERT phase III (12). A high-dose of very early mobilization within 24 h of stroke onset is not recommended by the AHA Stroke Guidelines of 2016 and 2019 (13, 14).

Since then, numerous trials have been investigating the effects of EM and VEM in stroke patients. Two recent systematic reviews by Langhorne et al. (15) and Rethnan et al. (16) investigated large trials of VEM and EM since 2008. The authors recommend that the commencement of mobilization should only be considered 24 h post-stroke, but the determination of the optimal dose of mobilization, as well as the identification of responders and non-responders to treatment remains unclear.

The acute phase of a neurological condition during hospitalization is timely to estimate the functional condition of these patients (17). The strength of muscles as hip extensors and trunk stabilizers (TWIST algorithm) seen in the MRC score can predict the need for assistance during walking 12 weeks post cerebral ischemia (17). The strength of hip abductors, ankle dorsiflexors, and trunk control (HAAD score) can predict good functional prognostics 90 days after hospital discharge (18). All of these groups of muscles usually are stimulated in activities performed in EM protocols.

EM protocols are generally composed of activities such as sitting out of bed, standing, and walking (9). In AVERT phase II (10), out-of-bed activities are stimulated in the hyperacute stroke phase and demonstrate the good feasibility and effectiveness of this intervention. AMOBES trial (11) compared passive mobilization and out-of-bed activities with resistive exercises in the trunk and limbs *plus* intensive task-oriented training commencing <72 h from a stroke. The authors found no change in the motor domain of the Fugl–Meyer score between both interventions.

Most of the motor activities presented in EM protocols can be measured by physiotherapists across diverse functional scales such as Physical Function in Intensive Care Test–scored (PFIT -s) (19), Chelsea Critical Care Physiotherapy Assessment Tool (CPAx) (20), Functional Status Score for the Intensive Care Unit (FSS ICU) (21), Intensive Care Unit Mobility scale (IMS ICU mobility scale) (22), John Hopkins Highest Level of Mobility (JH-HLM) (23) and a few others. However, these approaches have different sensitivities for predicting changes in patients' conditions (24). ICF, otherwise, is not a functional scale, it goes further by being a classification of body structures and activities-participation domains that can provide a universal language of integral health status in different rehabilitation settings. The performance qualifier in ICF might indicate the extent of a problem during the execution of specific activities in an individual's current environment (25).

In 2003 the World Confederation for Physical Therapy (WCPT) endorses ICF as one of the principal frameworks for recording outcomes in physical therapy practice. In a hospital environment, ICF has been widely explored and contemplates the most relevant patient conditions managed by physical therapists in the acute and post-acute care settings (1). Previous studies have gathered the most common ICF categories presented in neurological, cardiopulmonary, and musculoskeletal issues and validated them into Core Sets used in hospital settings (26, 27). Furthermore, there are current international efforts to implement ICF in acute rehabilitation, including the use of the ICF Generic-30 Set and the short version, ICF Generic-7 Set.

Implementation of ICF may help clinicians to broaden their perspectives regarding patient functioning (28). To rate the severity of a problem in ICF components, the WHO proposes a qualifier scale rating 0–4 (0 no problem to 4 severe problems).

This system of classification is known as a generic scale and can be used in conjunction with first, second, and third qualifiers. It is possible to compare data from before and after treatments in a biopsychosocial model, considering patients' physical functioning, participation ability, and the influence of environmental factors (29). Zhang et al. (29) observed positive correlations between ICF qualifiers in the perspective of body structure, activity-participation, and environmental components with clinical assessment instruments for stroke, including the Modified Barthel Index and National Institutes of Health Stroke Scale, both commonly used in a hospital environment.

In EM protocols applied in intensive or intermediate care settings, ICF can help detect patients' activity demands, and consequently, help professionals manage the best inpatient rehabilitation program.

The primary aim of the present study was to analyze the activity level of neurological inpatients in admission and delivery after a Neurological Early Mobility Protocol (NEMP) in intermediate care settings in a public hospital in Brazil using Activity Level categories, HPMQ, and MBI scores. The secondary outcome is to analyze the ICF performance qualifier, specifically in the activity domain, transposing HPMQ and MBI scores to the corresponding ICF performance qualifier.

## Materials and Methods

### Design Overview, Setting, and Participants

This is an international prospective study. The local Ethics and Research Committee has approved this research, with CAAE n 39932114.1.0000.5257 and Rebec: RBR-9n35t4. All patients or caregivers provided written consent regarding their participation in the study. Patients with neurological conditions were recruited between 2015 and 2018 during hospitalization in a public university in Rio de Janeiro, Brazil.

### Intervention

**Figure 1:**
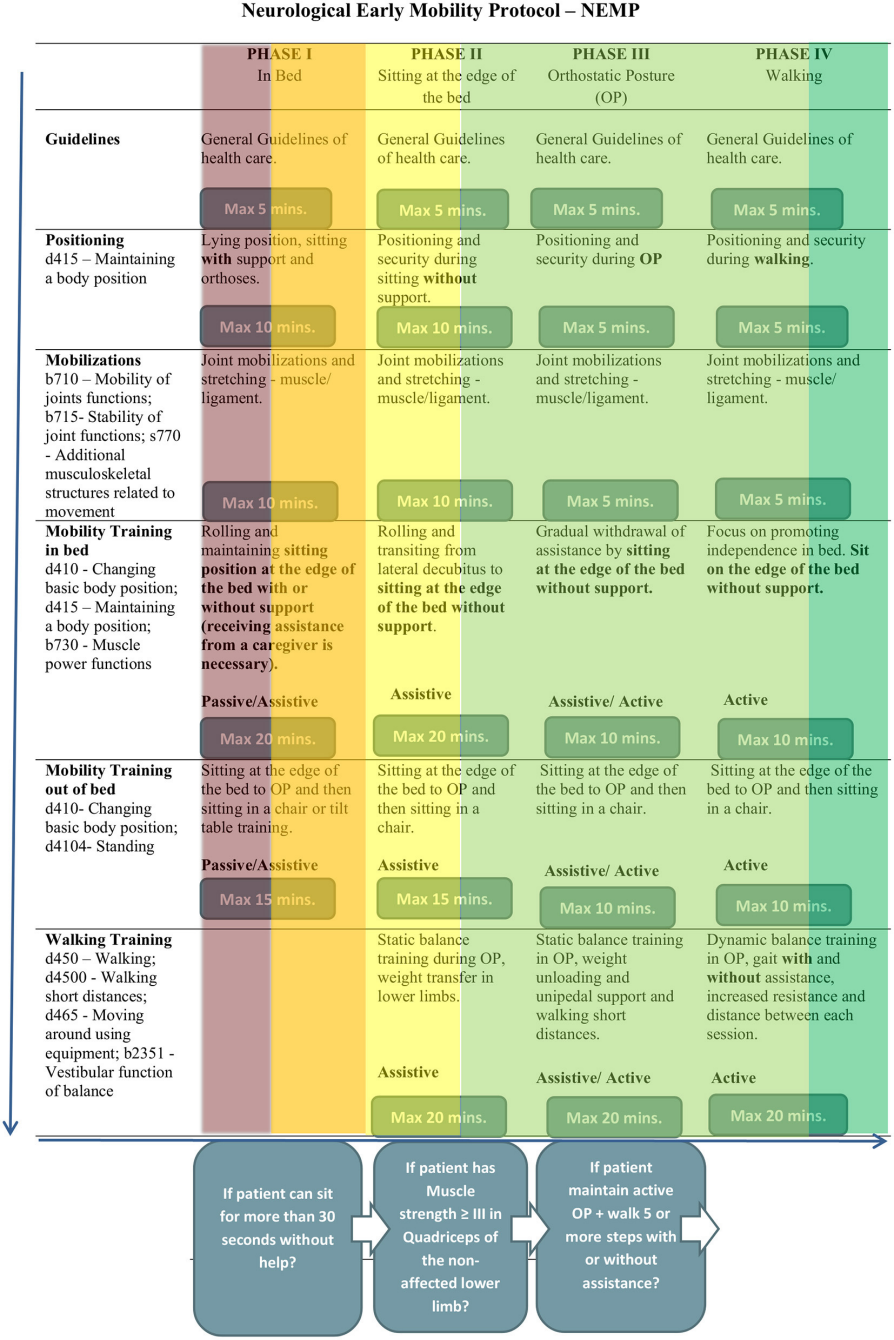
Neurological early mobility protocol–NEMP.

The NEMP (Figure 1) was adapted from Perme and Chandrashekar (7) and Drolet et al. (8) and consists of 65 exercises distributed along four progressive phases. Different from other EM protocols, it is directed to patients hospitalized in wards environments and not only indicated in acute unit care as stroke units, and helps multidisciplinary teams, especially physiotherapists, to program their rehabilitation approaches, according to a patient's functional status.

Each of the four phases has characteristics that correspond to a patient's motor activity level. Phase I is dedicated to patients who need to improve bed mobility, phase II helps patients to acquire independence while sitting at the edge of the bed, phase III promotes stability during upright position and phase IV aims to obtain safety in walking activity. In NEMP the term “mobility” concerns task-oriented exercises based on kinesiological principals and out-of-bed activities, while “early” means “optimal window of time” and then, the beginning of therapy as early as possible, considering a patient's clinical stability.

The admission to NEMP was a multidisciplinary team decision. To provide an accurate clinical indication, NEMP has *inclusion* and *exclusion criteria* (Table 1) (30) to ensure minimal adverse events. A patient would be indicating NEMP if they had systolic blood pressure between 90 and 160 mmHg, a heart rate of between 40 and 100 beats per minute, a respiratory rate of <25 breaths per minute, peripheral oxygen saturation >90%, and axillary temperature <38°C.

**Table 1:** Exclusion Criteria for NEMP.

**Exclusion criteria**	
**Respiratory criteria**	**Neurologic criteria**
Patients with Endotracheal tube	Deterioration of Neurological Status in the last few hours
Tracheostomy with PEEP >5 cm H_2_O	Intracranial pressure >20 mmHg
Tracheostomy with FiO_2_ >60%	Uncontrolled epilepsy
**Cardiovascular criteria**	**Hematologic criteria**
Need for vasopressors	Platelets <20 000
Unstable arrhythmia	Hematocrit <25%
Use of continuous vasodilator	Hemoglobin <7 g/dL
Recent myocardial infarction or angina	**Orthopedic Criteria**
Active bleeding	Acute Fracture in Lower Limbs
	Fracture or other instability of spine

*PEEP, Positive-End-Expiratory Pressure; FiO_2_, Fraction of Inspired Oxygen*.

NEMP has *interruption criteria* that allow the therapist to interrupt a session if the patient presents alert signs of changes in health status. Vital signs were evaluated during the entire session, and could be interrupted if the patient had important changes in heart rate, chest pain symptoms, oxygen saturation <88%; signs of respiratory discomfort; hypotension associated with fainting; falling during the session, and if the patient requests to stop.

Patients received treatment with NEMP five times a week until patients were discharged from physiotherapy.

### Outcome Measures

The primary outcome is to analyze the activity level of neurological inpatients in admission and delivery after a Neurological Early Mobility Protocol (NEMP) in intermediate care settings in a public hospital in Brazil using Activity Level categories, HPMQ, and MBI scores.

The secondary outcome is to analyze the ICF performance qualifier, specifically in the activity domain, transposing HPMQ and MBI scores to the corresponding ICF performance qualifier.

### Outcomes Assessments

#### Activity Level Categories

**Figure 2:**
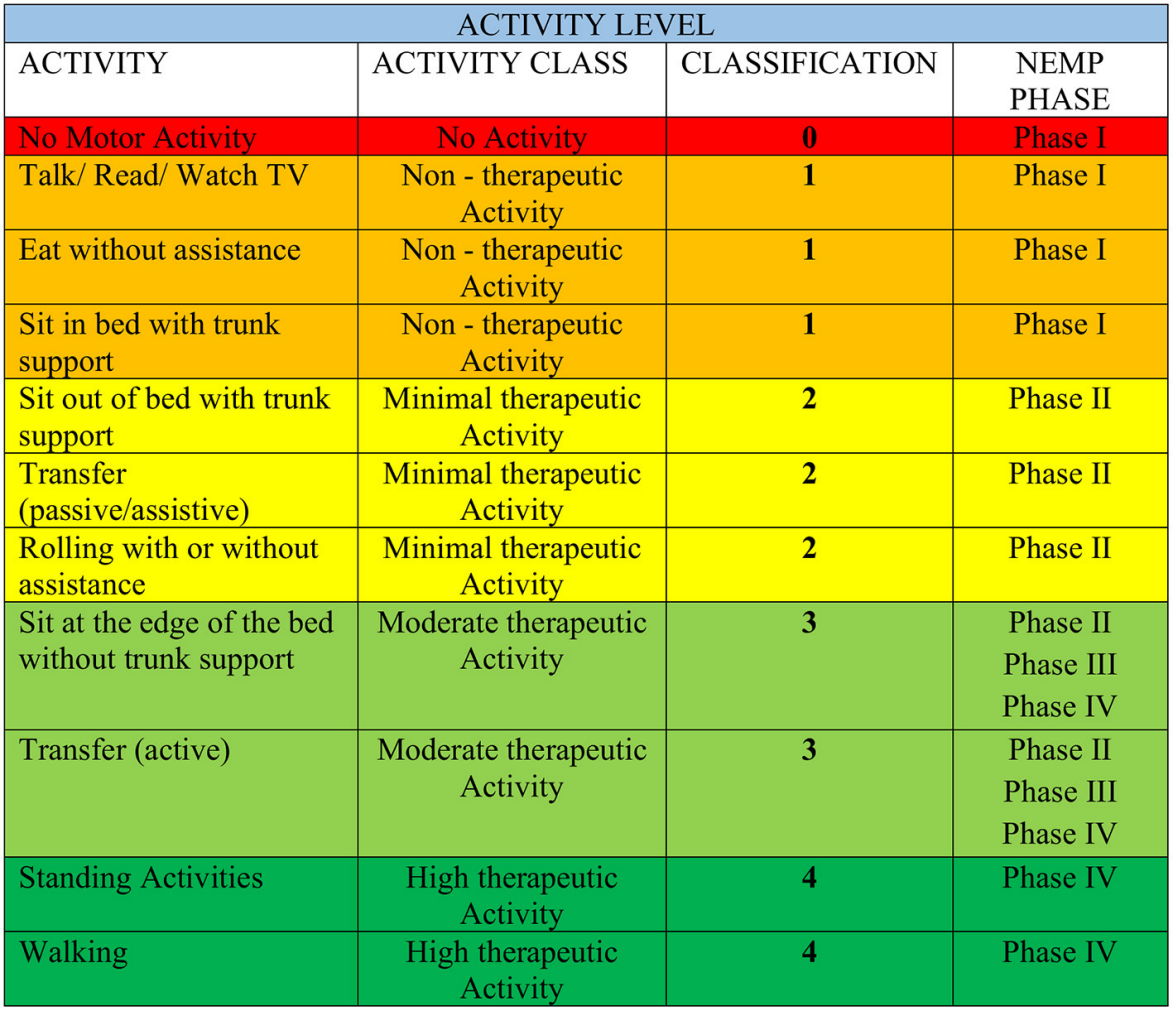
Activity Level category adapted from Bernhardt et al. (3). The colors organized in AL are in accordance with those in Figure 1 of the NEMP.

Initially, patients were categorized according to their Activity Levels [ALs–Figure 2—adapted from Bernhardt et al. (3)] to determine the NEMP phase to initiate the EM protocol. ALs were also evaluated in the first and last sessions of NEMP.

#### Level of Assistance in Hospital Activities According to HPMQ

**Figure 3:**
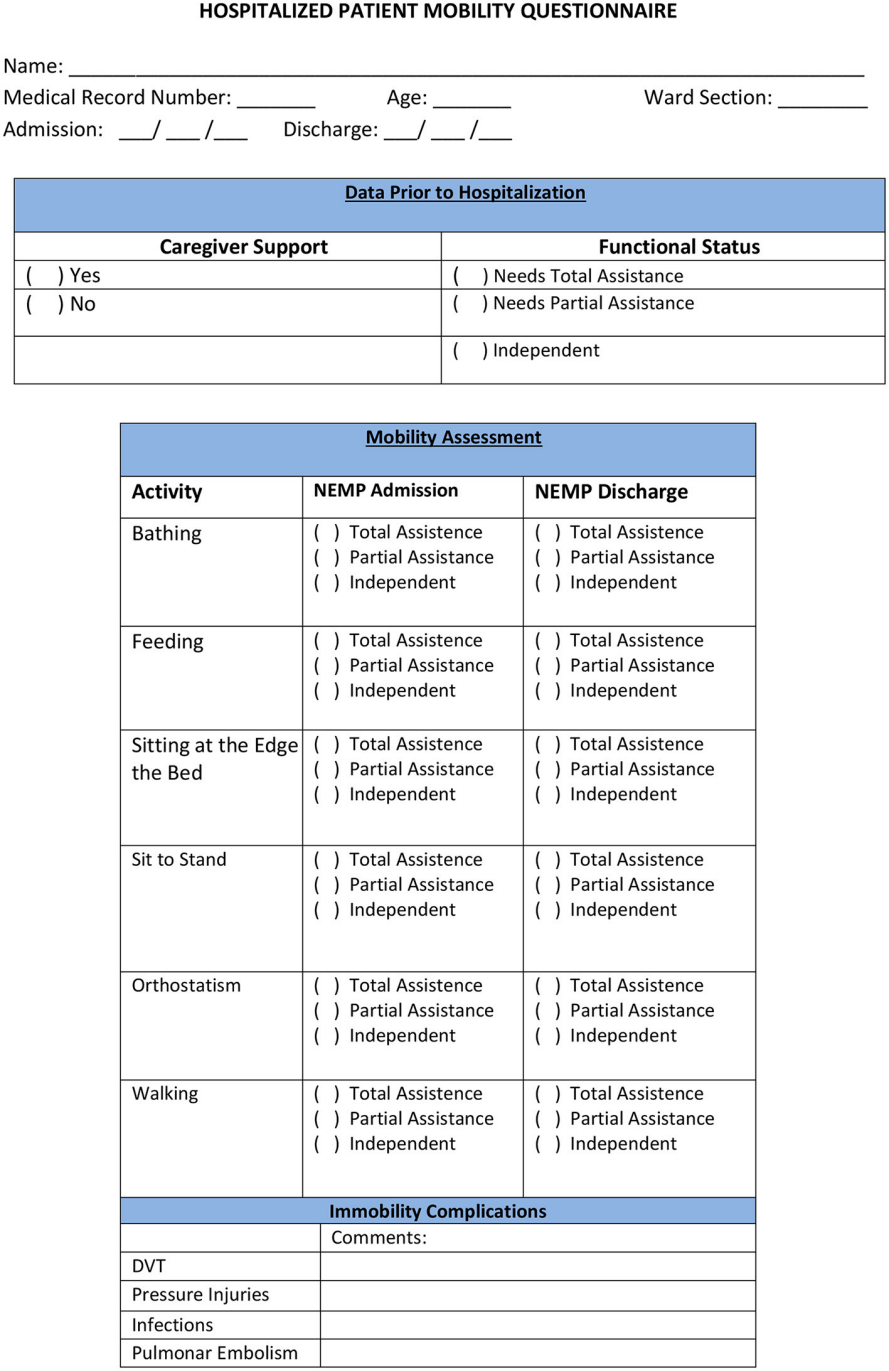
Hospitalized patient mobility questionnaire.

The Hospitalized Patient Mobility Questionnaire (HPMQ) was adapted from activity level categories from Bernhardt et al. (3) (HPMQ–Figure 3). This assessment was implemented to classify in scores patients' level of assistance during the performance of six hospital activities: bathing, feeding, sitting at the edge of the bed, changing from sitting to standing position, orthostatism, and walking. Each activity received three possible scores: 0 (total assistance), 1 (partial assistance), and 2 (independent), having a total score of 12 points which means that the higher the score, the higher the patients' independence level.

The scores obtained from each of the 6 activities analyzed in HPMQ were transposed to ICF first performance qualifier, which represents the extent of a problem in the Activity and Participation Component. To perform the ICF correlation, a score of 2 on the HPMQ scale was associated with 0 and 1 in ICF (mild and no problem). The score of 1 in HPMQ correlates to scores of 2 in ICF (moderate problem). The score 0 (total assistance) in the HPMQ was associated with scores 3 and 4 in ICF (severe and complete problem) (Table 2). The same process of recodification into ICF can also be seen in García et al. (31).

**Table 2:** Scores from HPMQ recoded in ICF performance qualifier.

**Score from HPMQ (each activity)**	**ICF Performance Qualifier**	**ICF Extent of the Problem**
0-total assistance	4	Complete and Severe problem
	3	
1-partial assistance	2	Moderate problem
	1	
2-independent	0	Mild and No problem

*HPMQ, Hospitalized Patient Mobility Questionnaire; ICF, International Classification of Functioning, Disability, and Health*.

The six activities present in HPMQ were linked with categories from the ICF activity domain and were analyzed with performance qualifiers (0–4): d510 bathing; d570 feeding; d4153 sitting at the edge of the bed (specification of d415: maintaining body posture); d4104 sit to stand (specification of d410: changing body posture); d4154 orthostatism (specification of d415: maintaining body posture); d450 walking activity [this process is in accordance with ICF linking roles from Cieza et al. (32)].

#### Level of Assistance in Activities of Daily Living According to MBI

As well as HPMQ, Modified Barthel Index (MBI) (33) was also applied at NEMP admission and discharge. A maximal numerical score of 100 can be obtained and is distributed along with five score intervals: ranges between 0 and 25 denote total dependency, 26–50 severe dependency, 51–75 moderate dependency, and 76–99 mild dependency. Considering the correlation between ICF and MBI (Table 3), a score of 100 in MBI was associated with 0 on the ICF generic scale (no problem). An MBI score between 76 and 99 was linked with 1 (mild problem–ICF), 51–75 with 2 (moderate problem–ICF), 26–50 with 3 (severe problem–ICF), and 0–25 with 4 (complete problem–ICF). MBI has ten activities based on the amount of physical assistance required to perform a task. The MBI total score was re-coded into ICF categories.

**Table 3:** Recoding MBI into ICF.

**MBI–score interval**	**MBI classification**	**ICF performance qualifier**	**ICF extent of the problem**
0–25	Total dependency	4	Complete problem
26–50	Severe dependency	3	Severe problem
51–75	Moderate dependency	2	Moderate problem
76–99	Mild dependency	1	Mild problem
100	Independent	0	No problem

*MBI, Modified Barthel Index; ICF, International Classification of Functioning, Disability and Health*.

The process of linking these two instruments with ICF followed the Linkage Rules suggested by Cieza et al. (32) to establish conceptual equivalence between the measures. All the meaningful concepts contained in the items of these two measures are linked to the ICF categories.

### Statistical Analysis

Analysis was performed using Statistical Package for Social Sciences software version 20.0 for Windows (IBM SPSS Statistics).

Descriptive analysis was used to determine the samples' characteristics regarding age, length of hospital stay, days to start NEMP, and the number of sessions were carried out using mean and standard deviation. Discrete variables such as the HPMQ and MBI scale scores, with non-normal distribution are reported as the median [Interquartile range] and the 95% CI.

Therefore, a nonparametric test was used. Comparative analyses between NEMP admission and discharge through HPMQ total score and for each of the six HPMQ activities, were performed by the Wilcoxon Signed Rank Test.

For MBI data, Wilcoxon Signed Rank Test also compared data from NEMP admission and discharge.

## Results

**Figure 4:**
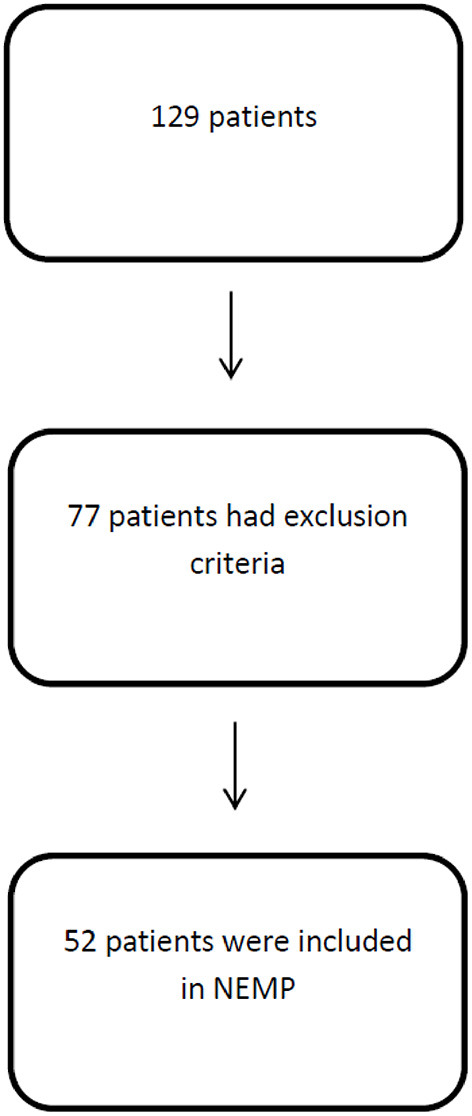
Flowchart with patients excluded and included in NEMP.

One hundred and twenty-nine patients were assessed by physiotherapists and 52 were eligible for NEMP after inclusion and exclusion criteria analysis (Figure 4).

Fifty-two patients were included with an age of 55 ± 20 (mean ± SD) years and a length of hospital stay of 33 ± 21 days. Fifty-two percent of patients were male and diagnostics varied between neuromuscular diseases (1), peripheral nerve injuries (5), and first motor neuron lesions (46). Comorbidities varied Systemic Arterial Hypertension (40%), Diabetes Mellitus (17%), Dyslipidemia (17%), and Atrial Fibrillation (14%). A major profile of patients is included in Table 4.

**Table 4:** Baseline characteristics of the treated population.

	**Mean ±SD**
Age (years)	55 ± 20
Length of hospital stay (days)	33 ± 21
Time since hospital admission until NEMP 1st session (days)	10 ± 9
Number of NEMP sessions	8 ± 5

*NEMP, Neurological Early Mobility Protocol*.

### Activity Level Data

Patients who were considered eligible for NEMP have their activity level classified into five categories AL 0-AL5. The initial AL determined the NEMP phase where the patient initiated the EM protocol.

Of all 52 patients, 6 (12%) were initially categorized as AL 0 and five (9%) continued as AL 0 at discharge. Only one patient in six (16%) at AL 0 improved to AL 4. At AL 1, there were 12 (23%) participants at the admission, the remaining six (12%) patients at AL 1 at discharge. Considering the initial 12 patients in AL 1, four (33%) progressed to AL 2 and two (17%) to AL 4.

Thirteen (25%) patients of 52 initiated NEMP as AL 2 category. This was the classification with more functional progress, constituting at discharge occasion eight (15%) patients. Of the initial 13 patients in AL 2, seven (54%) improved to AL 3, and two (15%) were discharged as AL 4. In NEMP admission, 10 (19%) patients composed AL 3 category and at discharge, there were 13 (25%) patients. From the initial 10 participants in AL 3, four (40%) changed to AL 4. There was no regression in any of the 11 participants (21%) classified as AL 4 at admission. Table 5 presents the percentage of patients in the first and last session of NEMP. Table 6 represents the migration of patients through AL categories.

**Table 5:** Activity Level (AL) of patients in NEMP admission and discharge.

**AL**	Number of patients at NEMP admission (%)*	Number of patients at NEMP discharge (%)*
AL 0	*n* = 6 (12%)	*n* = 5 (9%)
AL 1	*n* = 12 (23%)	*n* = 6 (12%)
AL 2	*n* = 13 (25%)	*n* = 8 (15%)
AL 3	*n* = 10 (19%)	*n* = 13 (25%)
AL 4	*n* = 11 (21%)	*n* = 20 (39%)

AL, Activity Level; NEMP, Neurological Early Mobility Protocol. *Consider % of total sample (n = 52).

**Table 6:** Percentage of Patients that Migrated through AL categories.

**AL**	**Percentage of Patients that migrated through AL**
AL 0 (*n* = 6)	16% to AL 4
AL 1 (*n* = 12)	33% to AL 2
	17% to AL4
AL 2 (*n* = 13)	54% to AL 3
	15% to AL 4
AL 3 (*n* = 10)	40% to AL 4
AL 4 (*n* = 11)	100% to AL 4

### HPMQ Data

Considering the total score of HPMQ (0–12), the group of 52 patients showed a median of 1 [IQR 4 (95% IC 1.4; 3.3)] in NEMP admission and 4 [IQR 6 (95% IC 3.8; 5.7)] in NEMP discharge (*p* < 0.05, Wilcoxon).

### ICF Performance Qualifier Through HPMQ Data

Progressions in ICF performance qualifier were observed in the activity of bathing that improved from severe to moderate problem ex.: d510.3 to d510.2; Sitting at the edge of the bed changed from moderate to mild problem ex.: d4153.2 to d4153.1; Sit to Stand raised from d4104.3 to d4104.2–severe to a moderate problem, as well as orthostatism ex.: d4154.3 to d4154.2. There were no changes in feeding (d570.2) and walking activities (d450.3) (Table 7).

**Table 7:** Descriptive outcomes from HPMQ in NEMP admission and discharge and comparison of these two time points through Wilcoxon Signed Rank Test, HPMQ re-codification into ICF performance qualifier.

	**Scores of HPMQ's activities (0–2)** **(*****n*** **=** **52)**								**Wilcoxon Signed Rank Test (discharge-admission) *p*-value**
**HPMQ**	**NEMP admission**				**NEMP discharge**				
	**25%**	**50%**	**75%**	**IQR (95% CI)**	**25%**	**50%**	**75%**	**IQR (95% CI)**	
Bathing	0	0	1	1 (0.27; 0.73)	1	1	2	1 (0.87; 1.24)	*p* < 0.05
Feeding	1	1	1	0 (0.92; 1.16)	2	1	2	1 (1.04; 1.35)	*p* < 0.05
Sitting at the edge of the bed	0	1	2	2 (0.62; 1.15)	1	2	2	1 (1.40; 1.79)	*p* < 0.05
Sit to stand	0	0	2	2 (0.50; 1)	0	1	2	2 (0.84; 1.35)	*p* < 0.05
Orthostatism	0	0	1	1 (0.21; 0.63)	0	1	2	2 (0.59; 1.07)	*p* < 0.05
Walking	0	0	0	0 (0.16; 0.57)	0	0	1	1 (0.44; 0.91)	*p* < 0.05

**Table:** 

**ICF PERFORMANCE QUALIFIERS**	**25%**	**50%**	**75%**		**25%**	**50%**	**75%**		
Bathing **d510**	**2**	**3**	**3**		1	**2**	**2**		
Feeding **d550**	**2**	**2**	**2**		**1**	**2**	**2**		
Sitting at the edge of the bed **d4153**	**1**	**2**	**3**		**1**	**1**	**2**		
Sit to stand **d4104**	**0**	**3**	**2**		**0**	**2**	**2**		
Orthostatism **d4154**	**2**	**3**	**3**		**1**	**2**	**3**		
Walking **d450**	**3**	**3**	**3**		**2**	**3**	**3**		

*Data considering (n = 52)*.

*HPMQ, Hospitalized Patient Mobility Questionnaire; NEMP, Neurological Early Mobility Protocol; Percentiles 25, 75, and median of each HPMQ activity; p-value line refers to the differences between HPMQ scores in NEMP admission and discharge through Wilcoxon Signed Rank Test. Bold values means p < 0.05*.

### MBI Scale

Scores obtained from MBI (0–100 score) revealed a median of 36 [(IQR−35. (95% CI 31.5; 41.1)] on NEMP admission and 52 [IQR−50 (95% CI 43.2; 60.3)] on discharge (p = 0.001, by Wilcoxon Signed Rank test) describing improvements from Severe Dependence to Moderate Dependence.

### ICF Performance Qualifier Through MBI

Transposing scores from MBI into ICF performance qualifiers, outcomes at admission had a median of 36 (severe dependency in MBI, 26–50) corresponding to ICF severe problem (3) and at discharge BMI median score of 52 (moderate dependency in MBI, 51–75) corresponding to ICF moderate problem (2).

### HPMQ Data Considering AL Categories

Patients were distributed among the five ALs group categories. After NEMP intervention we detected no significant change in any activity from HPMQ in AL 0. Otherwise, patients from AL 1 had improvement for sitting at the edge of the bed with median [IQR (95% CI)] of 0 [0 (0.09; 0.45)] at admission to 1 [1 (0.62; 1.56)] at discharge occasion (*p* = 0.01).

Of all AL categories, AL 2 was the most responsive to NEMP. There were favorable outcomes for bathing 0 [0 (−0.29;0.24)] to 1 [0 (0.75;1.25)]; (*p* = 0.01), sitting at the edge of the bed 0 [2 (0.01; 1.07)] to 2 [0 (1.41;2.13)]; (*p* = 0.004), transition from sitting position to standing 0 [1 (−0.01; 0.78)] to 1 [2 (0.56; 1.60)]; (*p* = 0.01) and orthostatism 0 [0 (−0.09; 0.24)] to 1 [1 (0.24; 1.15)]; (*p* = 0.03). This group was treated with NEMP phase II exercises, receiving trainings that invest in trunk stability, which is fundamental to reach independence in most of this improved HPMQ items.

AL 3 represents the patients who can sit at the edge of the bed without trunk support and complete active transfers. They developed changes in bathing 0 [1 (−1; 0.9)] to 1 [1 (0.95;1.65)]; (*p* = 0.01), orthostatism 0 [1 (−0.05;0.6)] to 1 [2 (0.33;1,67)]; (*p* = 0.03) and walking 0^*^ / 1 [2 (0.14;1.46)]; (*p* = 0.05) after receiving sessions with NEMP phase II, III or IV.

AL 4 (treated with NEMP phase IV) only expressed improvements for feeding activities, the only item in HPMQ that had no statistical significance in none of the other categories.

### ICF Performance Qualifier Through HPMQ Considering AL Categories

ICF performance qualifier changed for all AL groups for bathing activity, with the exception of AL 4. In the feeding task, even though the progression for AL 4 group, when transposing to ICF, none of the ALs expressed improvements.

Advances in performance qualifiers were observed in AL 2 for the same activities with a significant statistic in the analysis shown above. They include bathing (changed from d510.3 to d510.2), sitting at the edge of the bed (d4153.3 to d4153.1), sit to stand (d4104.3 to d4104.2), and orthostatism (d415.3 to d415.2). In AL 3, only bathing and orthostatism improved, with the performance qualifier changing from d510.3 to d510.2 and d415.3 to d415.2, respectively. AL 4 did not express any improvements in the ICF performance qualifier.

The outcomes relating to HPMQ data and respective ICF Performance Qualifiers are described in Table 8.

**Table 8:** Descriptive outcomes from HPMQ in NEMP admission and discharge, their re-codification into ICF performance qualifier, and comparison of these two time points through Wilcoxon Signed Rank Test. Data from each AL group.

	**AL 0 (*n* = 6)**	**AL 1 (*n* = 12)**	**AL 2 (*n* = 13)**	**AL 3 (*n* = 10)**	**AL 4 (*n* = 11)**
	**Median [IQR (95% CI)] Admission/Discharge**	**Median [IQR (95% CI) Admission/Discharge**	**Median [IQR (95% CI)] Admission/Discharge**	**Median [IQR (95% CI)] Admission/Discharge**	**Median [IQR (95% CI)] Admission/Discharge**
Bathing	0 [0 (−0.26; 0.6)]/1 [1 (−0.07; 1.2)]	0 [0 (−0.22; 0.59)]/0 [0 (−0.22; 0.59)]	0 [0 (−0.29;0.24)] /1 [0 (0.75;1.25)]	0 [1 (−1; 0.9)] /1 [1 (0.95;1.65)]	2 [0 (1.09; 2.18)]/2 [0 (1.55;2.09)]
*p*-value	0.15	0.2	**0.01**	**0.01**	0.4
ICF d510	3/2	3/2	3/2	3/2	1/1
Feeding	1 [1 (0.12; 1.21)]/1 [1 (0.12; 1.21)]	1 [0 (0.71; 1.11)]/1 [0 (0.71;0.11)]	1 [0 (0.76; 1.09)] /1 [0 (0.75;1.25)]	1^*^/1 [1 (0.95;1.65)]	2 [1 (1.19; 1.9)]/2 [0 (1.71; 2.11)]
*p*-value	1	1	0.3	0.08	**0.04**
ICF d550	2/2	2/2	2/2	2/2	1/1
Sitting at the edge of the bed	0 [1 (−0.52; 1.1)]/1 [2 (0.06; 1.94)]	0 [0 (0.09; 0.45)] /1 [1 (0.62; 1.56)]	0 [2 (0.01; 1.07)]/2 [0 (1.41;2.13)]	2 [1 (0.8; 2)]/2 [0 (1.35; 2.25)]	2 [0 (1.71; 2.11)]/2
*p*-value	0.1	**0.01**	**0.004**	0.1	0.3
ICF d4153	3/2	3/2	3/1	1/1	1/1
Sit to stand	0^*^/0 [1 (−0.52; 1.19)]	0 [0 (−0.09; 0.45)]/0 [−0.16; 0.71)]	0 [1 (−0.01; 0.78)] /1 [2 (0.56; 1.60)]	1 [2 (0.42; 1.58)]/1 [1(0.9;1.9)]	2^*^/2^*^
*p*-value	0.31	0.18	**0.01**	0.15	1
ICF d4104	3/3	3/3	3/2	2/2	1^*^/1^*^
Orthostatism	0^*^/0 [1 (−0.21; 0.88)]	0^*^/0 [0 (−0.11; 0.29)]	0 [0 (−0.09; 0.24)]/1 [1 (0.24; 1.15)]	0 [1 (−0.05;0.6)] /1 [2 (0.33;1,67)]	1 [0 (1.1; 2.09)] /2 [0 (1.55; 2.09)]
*p*-value	0.15	0.15	**0.03**	**0.05**	0.3
ICF d4154	3/3	3/3	3/2	3/2	1/1
Walk	0^*^/[1 (−0.26; 0.60)]	0^*^/0 [0 (−0.09; 0.45)]	0 [0 (−0.09; 0.24)] /0 [1 (−0.01; 0.78)]	0^*^/1 [2 (0.14;1.46)]	2 [1 (1.41; 2)]/2 [1 (1.41; 2.04)]
*p*-value	0.3	0.15	0.15	**0.03**	0.5
ICF d450	3/3	3 /3	3/3	3/3	1/1

*HPMQ, Hospitalized Patient Mobility Questionnaire; NEMP, Neurological Early Mobility Protocol; p-value line refers to the differences between HPMQ scores in NEMP admission and discharge*.

* *Some HPMQ outcomes were constant, having no difference between admission and discharge. Bold values means p < 0.05*.

### MBI Data Considering AL Categories

Considering each AL group (Table 8), MBI showed significant improvements for ALs 1, 2 and 3, but with no difference for ALs 0 and 4. AL 1 improved from 20 [IQR−18 (95% CI 14.14; 27.03)] to 29 [IQR−31 (95% CI 17.60;46.13)] points, changing from Total Dependency to Severe Dependency (*p* = 0.01), AL 2 from 37 [IQR −24 (95% CI 26.39; 44.53)] to 51 [IQR−24 (95% CI 40.10; 65.13)]–Severe Dependency to Moderate Dependency (*p* = 0.01), as well as AL 3, that also improved from 44 [ IQR−25 (95% CI 34;35)] to 64 [IQR−32 (95% CI 50.53; 73.27)] points–Severe Dependency to Moderate Dependency (*p* = 0.01).

### ICF Performance Qualifier Through MBI Scale Considering AL Categories

The ICF performance qualifiers obtained from MBI progressions were observed for AL 1 (advanced from complete to severe problems), AL 2 and 3 (both improved from severe to moderate problems). MBI outcomes re-coded to ICF considering the five ALs groups are described in Table 9.

**Table 9:** Median [IQR (95% CI)] scores of MBI.

	**MBI Admission**	**ICF Admission**	**MBI Discharge**	**ICF Discharge**	**MBI *p*-value Wilcoxon Signed Rank Test**
AL 0	0 [1 (95% CI 0.5; 1.1)]	4	1 [12 (95%CI−6,51; 19.18)]	4	0.18
AL 1	20 [18 (95% CI 14.14; 27.03)]	4	29 [31 (95% CI 17.60; 46.13)]	3	**0.01**
AL 2	37 [24 (95% CI 26.39; 44.53)]	3	51 [24 (95% CI 40.10; 65.13)]	2	**0.01**
AL 3	44 [25 (95% CI 34;35)]	3	64 [32 (95% CI 50.53; 73.27)]	2	**0.01**
AL 4	95 [17 (95% CI 70.32; 98.22)]	1	95 [18 (95% CI 78.97; 97.40)]	1	0.08

*p-value column refers to the differences between MBI scores contrasting NEMP admission and discharge. MBI score interval: (0–25)–total dependency; (26–50)–severe dependency; (51–75)–moderate dependency; (76–99)–mild dependency; (100) – Independent. Bold values means p < 0.05*.

## Discussion

To make inferences in the ICF activity domain after a Neurological Early Mobility Protocol in an inpatients cohort, we analyzed three different evaluations: a) activity level (AL) to perform hospitals activities (5 categories); b) HPMQ which evaluates the level of assistance to perform hospitals activities (0–12); c) MBI classify the level of assistance of daily living Activity (0–100).

This study found that most neurologic patients in a public hospital in Brazil (Rio de Janeiro city), had low activity levels during hospital stay before an EM protocol. In all AL categories patients reached higher AL categories, except for AL 4, the most independent level. In AL 0, one patient had a potential improvement receiving discharge as AL4. In summary, categories with lower AL such as 0, 1, and 2 have lost their patients to the more independent groups, such as AL 3 and 4.

This data indicates that patients with neurological conditions might be responsive to NEMP, even though they have started the EM protocol out of the prescribed window observed in the literature (24–72 h). The same perspective can be represented with HPMQ scores, which revealed a statistically significant difference when comparing these two time points. The MBI scale also indicated changes in the construct as Severe Dependence improved to Moderate Dependence after intervention with NEMP.

Respecting the biomedical model, which is fixed in a specific diagnostic, the International Classification of Disease (ICD–WHO) does not change during the rehabilitation process. On the other hand, ICF will reflect functional status according to the different aspects of a patient's health. In our study, ICF showed improvements through performance qualifiers when recoded from HPMQ and MBI. Patients had qualifiers predominantly such as 2 and 3 (moderate to severe problems) at NEMP admission (data from HPMQ), which denotes moderate and severe problems. At discharge, qualifier 2 was the most common. This classification encourages the fact that the ICF performance qualifier might be useful to describe changes in the domain of activity, before and after interventions. The changes in (0–4) ICF scores were congruent with improvements observed in HPMQ. In orthostatism and walking items, for example, changes from severe problems to moderate problems were observed, demonstrating that NEMP can help patients to perform both tasks with less assistance.

In the present study, we used ICF Linking Rules from Cieza et al. (32) to recode activities from two instruments of functional measure into ICF. This linking process is important in allowing researchers to compare meaningful concepts contained in measure items more faithfully. Similarly, Prodinger et al. (34) worked to establish the equivalence of scores obtained from the FIM and MBI through ICF chapters of Mobility (d4) and Self-care (d5). The authors showed that the ICF Linking Rules could establish conceptual equivalence between these 2 scales through the application of ICF. In trials by García et al. (16), commonly used scales to measure walking activity post stroke were re-coded in ICF qualifiers. The trial demonstrated that the ICF generic score can be sensitive at detecting changes between the pre and post rehabilitation program directed to chronic stroke.

Besides starting after the interval considered as “early” in EM (first 24–72 h), patients were sensitive to NEMP. In the literature, the time to initiate EM usually varies from the first 24 to 72 h after ICUs admission or from stroke (10, 11). We implemented NEMP in ward settings, where physiotherapists are usually requested through a physician order before initiating rehabilitation. This delay is not inherent to the present study, Indredavik et al. (35), for example, observed that there was a delay of 2–3 days before the patient received the first physiotherapy/mobilization training in global wards, in comparison to stroke units. Despite the delay, NEMP was useful for improving the activity levels of inpatients in wards settings.

In the current study, data showed AL 2 as the most sensitive to NEMP intervention, especially for bathing, sitting at the edge of the bed, and sitting to stand transition in HPMQ activities. In MBI scores, patients in AL2 improved from severe to moderate dependency. This group was initially conducted to phase II of NEMP, where most exercises recruit the muscles of the trunk, facilitating the independence for these tasks. In the ICF performance qualifier, we observed improvements for the same activities of HPMQ in AL 2.

Categories as AL 1 and 3 exhibited progressions in constructs of MBI and the correspondent ICF performance qualifier. The AL 1 group showed significant statistical improvement when comparing pre and post NEMP intervention, with changes in total dependency construct to severe dependency in MBI (ICF qualifiers 4–3). In ICF data transposed from HPMQ in AL 1, bathing (3 to 2) and sitting at the edge of the bed (3 to 2) activities revealed favorable outcomes. AL 3 changed from severe to moderate dependency in MBI and correspondent changes in 3 to 2 in ICF. Recoding HPMQ scores into ICF, we had better qualifiers for bathing and orthostatism (both 3 to 2).

For patients in AL 4 group, there was no difference between the admission and discharge of NEMP in the MBI scale or HPMQ, except for the feeding item. There were no changes in ICF recodification from these scales, either. AL4 is characterized by a higher independence level, and they might have already reached the top of their functionality, with no changes in outcome. AL 0 (correspondent to phase I in NEMP) only had favorable outcomes for ICF performance qualifier for bathing and sitting at the edge of the bed, which is a favorable outcome as this latter activity is a criterion for progression to phase II in NEMP.

Rating the severity of functioning problems has been a challenge in the clinical field. Uhlig et al. (36) reported low reliability while examining the interrater reliability of clinician ratings using ICF qualifiers and the ICF core set for rheumatoid arthritis. Using a 0–4 qualifier rating scale alone, there is no guarantee that the scores have the same meaning among the various raters. For example, the problems in category b280 (sensation of pain) have different aspects as the intensity of pain, frequency, and the site of pain that can be interpreted differently among raters (28). Recoding ICF qualifiers through validated scales might be a good strategy to minimize different possibilities of rating 0–4 ICF scores.

The two instruments used in the current trial measure the level of assistance during the performance of activities also presents in chapters of Mobility and Self-care in ICF from WHO (25). The difference between both scales is that HPMQ analyzes activities commonly performed in a hospital environment, and MBI assesses the activity of daily lives. Furthermore, the HPMQ scale used “sitting at the edge of the bed,” “sit to stand” transition, and “orthostatism,” activities that are not inserted in MBI. These tasks are usually trained in EM protocols (7, 8, 10) so that inpatients can improve independence during important activities before being discharged home.

It is noteworthy that using the ICF improves communication between interdisciplinary teams from different fields, helping professionals undertake more holistic thinking when elaborating treatment programs (29). In the current study, the research implication is that NEMP might be useful in improving the activity levels of patients with neurological conditions, hospitalized in the wards of a public university hospital in Brazil. We hope that using the ICF first performance qualifier through recodification of validated scales will strengthen the applicability of ICF.

## Limitations

The delay in initiating NEMP compared to the period observed in the literature is considered a limitation, as well as the absence of a control group to compare data and the fact that the study was carried out at only one center.

## Conclusion

Neurological patients hospitalized in wards have a low level of activity. In this study, data revealed an improvement in patients' AL after NEMP even if it was initiated after 72 h from hospital admission. The literature strongly recommended that EM protocols must be initiated within 48 h in stroke and other neurological disorders. This indicates a new time window for early rehabilitation in ward settings. NEMP reflected an alternative treatment that, despite being based on studies done in specialized ICUs and stroke units, revealed gains in mobility during hospitalization in wards.

## Data Availability Statement

The raw data supporting the conclusions of this article will be made available by the authors, without undue reservation.

## Ethics Statement

The studies involving human participants were reviewed and approved by Hospital Universitário Clementino Fraga Filho-UFRJ CAAE 39932114.1.0000.5257. The patients/participants provided their written informed consent to participate in this study.

## Author Contributions

FL, AF, and IB contributed to the conception, design, and analysis of data for the work. VC contributed with data collection. FL wrote the manuscript and performed data analysis. All authors agree with the submission of this study.

## Funding

This work was supported by the Higher Education Personnel Improvement Coordination (CAPES-Brazil).

## Conflict of interest

The authors declare that the research was conducted in the absence of any commercial or financial relationships that could be construed as a potential conflict of interest.

## Publisher's note

All claims expressed in this article are solely those of the authors and do not necessarily represent those of their affiliated organizations, or those of the publisher, the editors and the reviewers. Any product that may be evaluated in this article, or claim that may be made by its manufacturer, is not guaranteed or endorsed by the publisher.
